# Cognitive Mechanisms of Referential Ambiguity Resolution in L2 Russian by Chinese Learners: Evidence from Eye-Tracking

**DOI:** 10.3390/jemr19030063

**Published:** 2026-06-03

**Authors:** Tian Ran, Lijun Guo, Hong Xu

**Affiliations:** 1 School of Russian and Eurasian Studies, Shanghai International Studies University, 1550 Wenxiang Road, Shanghai 201620, China; 0234101603@shisu.edu.cn; 2 School of International Studies, Sun Yat-sen University, Guangzhou 510275, China; guolj5@mail.sysu.edu.cn

**Keywords:** referential ambiguity resolution, eye-tracking, cue weighting, typological distance, second language acquisition, L2 Russian, Chinese learners, morphosyntactic processing

## Abstract

A central question in second language (L2) sentence processing concerns how learners resolve referential ambiguity in real time, particularly when cues from their first language (L1) conflict with those of the target language. Given the substantial typological distance between Chinese (an analytic language) and Russian (a highly inflectional language), this study employs eye-tracking methodology to investigate the developmental trajectory of the cognitive mechanisms in referential ambiguity resolution among Chinese learners of Russian. The results revealed proficiency-related differences in ambiguity processing. Both proficiency groups showed increased processing difficulty when encountering ambiguous pronouns, indicating that referential ambiguity imposed a measurable online cost. High-proficiency learners read more efficiently overall, whereas low-proficiency learners showed a stronger first-mention anchoring pattern. These findings suggest that increasing L2 proficiency is associated with changes in processing efficiency and cue weighting during referential resolution. Notably, even high-proficiency learners did not categorically rely on Russian gender agreement to resolve reference in the morphologically disambiguated condition, suggesting that the integration of morphosyntactic cues into real-time reference resolution remains effortful at advanced proficiency. The study contributes eye-tracking evidence on how Chinese-speaking learners manage referential ambiguity in a morphologically rich L2.

## 1. Introduction

The resolution of referential ambiguity is central to comprehension. This process is not a straightforward matching operation but rather a dynamic weighting of multiple linguistic cues. These competing cues are distributed across multiple levels of language processing. Comprehenders draw on a variety of cues, such as the first-mention priority [1,2], subject priority [3,4,5], the Principle of Grammatical Parallelism [6,7,8], the thematic role of the referent and the temporal structure of the event [9,10], discourse coherence [11,12,13,14], and the distance to the antecedent [15,16]. These cues, originating at different representational levels, do not always converge. The competition among them constitutes a central issue in the study of anaphora resolution and offers a critical window into the underlying mechanisms of language comprehension.

While the mechanisms of referential ambiguity resolution have been extensively studied in analytic languages, the issue of cue competition in Russian, a language with rich inflectional morphology and flexible word order, remains an open question. Russian word order can serve information-structural and pragmatic functions, so cognitively prominent syntactic positions, such as the sentence-initial position, are not always occupied by the subject. At the same time, Russian relies on case, gender, and number agreement to mark grammatical functions and establish anaphoric dependencies. These morphological cues are highly reliable, and their weighting in processing by Russian native speakers can even surpass that of positional cues [17].

(1) Uchitel’ otkryl tetrad’ i uvidel, chto uchenik na ekzamene poluchil tol’ko dvoyku. Mozhet byt’, on bol’she vremeni udelit matematike.

(The teacher-NOM opened the notebook and saw that the student-NOM on the exam received-MASC only a ‘D’ grade. Maybe he-NOM will dedicate more time to mathematics).

For a Russian complex sentence like example (1), where the main clause and the subordinate clause each contain a potential antecedent, anaphor resolution in the subsequent sentence becomes a complex problem. In the second sentence, the referent the pronoun *on* (‘he’) is ambiguous: it could refer either to the subject of the main clause, ‘the teacher’ (*uchitel’*), or to the subject of the subordinate clause, ‘the student’ (*uchenik*). In such cases, the resolution mechanism is influenced by multiple competing cues—including those that are syntactic, semantic, pragmatic, and information-structural cues. The interplay of these factors during real-time processing has not yet been sufficiently investigated, e.g., [18].

## 2. Theoretical Models of Referential Ambiguity Resolution

Referential choice is a complex process. From the perspective of syntactic roles, the Subject Priority principle, e.g., [19] predicts that the pronoun *on* in sentence (1) should refer to the grammatical subject of the main clause, ‘the teacher’ (*Uchitel’*). However, this prediction is challenged by factors at the cognitive and semantic levels. First, from the standpoint of event structure and semantic roles, although ‘the teacher’ is the Experiencer of the overall perceptual event, ‘the student’ is the central participant in the event’s core content—namely, ‘receiving a D’. According to the effect of event temporal structure [10], an entity at the endpoint or in the Goal position of an event (‘the student’ in this case) possesses greater cognitive salience than an entity at the starting point (‘the teacher’). Similarly, expectation-based approaches to sentence processing [20] propose that a comprehender’s attention is drawn more to the outcome of an event and its core participants, thus favoring the resolution of the pronoun *on* to ‘the student’. Finally, from the perspective of discourse coherence, both interpretations appear plausible: resolving *on* to ‘the student’ (because he performed poorly, he needs to spend more time studying) can establish a Causal relation, whereas resolving it to ‘the teacher’ (because the student performed poorly, he needs to spend more time tutoring) can establish an Elaboration relation. This ambiguity requires the comprehender to engage in deeper pragmatic reasoning to ultimately determine the most likely referent.

## 3. Referential Ambiguity Resolution in Second Language Acquisition

How second language (L2) learners weigh and use these referential cues is a core cognitive puzzle in the field of L2 processing [21,22,23]. There is evidence suggesting that L2 learners can successfully acquire and apply the interpretation preferences of the target language, exhibiting processing patterns similar to those of native speakers, e.g., [24,25]. However, a substantial body of research indicates that even highly proficient L2 users often exhibit indeterminacy and processing difficulties when they must integrate multiple competing cues from syntax, semantics, and discourse in real time, e.g., [26,27,28].

In Generative Grammar theory, the concept of an ‘interface’ originally referred to the two levels of linguistic representation that connect the core computational system with external cognitive systems: Logical Form (LF) and Phonetic Form (PF) [29]. Subsequent research, particularly in Second Language Acquisition, has extended this notion to the levels at which grammatical modules interact with other cognitive domains, such as the syntax-semantics, syntax-morphology, and syntax-discourse interfaces, e.g., [27,30,31,32,33]. Interfaces are typically divided into two types: internal and external. The former refers to “the interface between narrow syntax and other linguistic modules (phonology, morphology, semantics),” whereas the external interface refers to “the interface of syntax with other cognitive modules” [34]. In other words, the internal interfaces concern the formal properties and computations of the linguistic system itself, while the external interfaces involve the integration of linguistic structure with higher-level cognitive domains such as discourse and pragmatics.

Regarding the acquisition difficulty of these two types of interfaces, it is widely held that external interfaces pose a greater challenge for L2 learners than internal interfaces. As Sorace [27] points out: “linguistic structures at the interface between syntax and other cognitive domains are less likely to be fully acquired than structures that do not involve this kind of interface.” This claim has received extensive empirical support across a range of various linguistic phenomena. For instance, Tsimpli and Sorace [31] found that L2 learners could master knowledge at the syntax-semantics interface (an internal interface) but were particularly vulnerable when processing knowledge at the syntax-discourse interface (an external interface). Similarly, Nossalik [35] reported that within the syntax-semantics interface (e.g., aspectual coercion), learners could acquire purely morphological rules, yet their processing speed slowed considerably when confronted with structures requiring the integration of pragmatic reasoning. Evidence from the semantics–pragmatics interface provides a comparable pattern. Feng [36], in a study on presuppositions, found that while L2 learners of English could compute the basic presupposition triggered by the verb *stop*, they were significantly less likely than native speakers to suspend this presupposition in contexts where such suspension was pragmatically required. This difficulty in inhibiting a default pragmatic inference further underscores the vulnerability of external interfaces.

For second language users, the real-time integration of referential cues in the target language is a well-documented challenge, particularly at the “interfaces” where multiple sources of information, including syntax, discourse, and pragmatics, must be jointly evaluated. Even advanced learners often exhibit processing difficulties in these domains [37]. According to the Interface Hypothesis, such difficulty arises from the considerable processing demands associated with coordinating information from distinct cognitive modules. This challenge is especially pronounced for native Chinese-speaking learners, for whom L1 transfer effects appear to be “both stronger and more persistent” than for learners from many other linguistic backgrounds.

First, in spoken Mandarin Chinese, the third-person pronouns (*tā*, 他, ‘he’; *tā*, 她, ‘she’; *tā*, 它, ‘it’) are homophonous, meaning that gender features are entirely unmarked phonologically. Second, as a pro-drop language, Chinese frequently employs null pronouns, rendering the use of overt pronominal forms less frequent and less obligatory than in languages such as English. This L1 profile may reduce learners’ tendency to automatically attend to morphological information, including gender, for interpretive purposes. Consequently, the gender errors made by Chinese learners when processing L2 pronouns are not only more frequent than those reported for Dutch-English or Spanish-English bilinguals [38,39], but they also persist even at higher proficiency levels, unlike what is observed among learners with a German background [40]. Therefore, when learning a morphologically rich language such as Russian, Chinese-speaking learners are more inclined to rely on position-based strategies grounded in their L1, word order, and preferences, and may be slower or less sensitive in employing the morphosyntactic cues that are critical for anaphora resolution in the target language.

Currently, the number of online processing studies specifically targeting anaphora resolution in L2 Russian is limited, yet the available findings reveal a complex interaction between L1 transfer and properties of the target language. On the one hand, research demonstrates that certain reliable morphological cues in Russian are learnable. For example, Kempe and MacWhinney [17] showed that although the Russian case system is more complex than the German one, native English speakers acquired Russian case marking faster than learners of German. They attributed this pattern to the very high “cue validity” of Russian morphological cues—that is, their consistency and frequency in the input—which facilitates the formation of input-based mappings.

However, on the other hand, L2 learners encounter substantial difficulty when morphological cues (such as grammatical gender), although formally unambiguous, must be integrated in real-time with discourse-level cues, including antecedent salience. Cho and Slabakova [41] reported that learners of Russian from both English and Korean backgrounds struggled with information structural phenomena, such as the expression of definiteness through word order variation. A similar pattern was found by Slioussar and Harchevnik [42] among native Chinese-speaking learners of Russian. When processing non-canonical Russian word orders, these learners showed significantly slower online reading times and lower offline comprehension accuracy than native speakers. Although they were able to follow broad discourse principles such as “the tendency to present given-information-before-new” information, they were not sensitive to more subtle, language-specific pragmatic constraints. Taken together, these findings suggest that while L2 learners can acquire robust and transparent morphological cues in Russian, their difficulties become more pronounced, and L1 transfer effects more persistent, when successful interpretation depends on the integration of discourse cues, information structure, and pragmatic context.

In summary, Chinese-speaking learners face distinctive challenges when processing morphologically rich languages due to the typological properties of their L1. At the same time, the acquisition of L2 Russian involves a dynamic negotiation between L1-based strategies and the robust cues provided by the target language. However, previous research has either relied on offline methods, which cannot capture the real-time dynamics of processing, or has focused on other language pairings and therefore does not illuminate the cognitive conflict specific to the Chinese–Russian combination, a pair marked by fundamental morphosyntactic differences. In particular, when the position-based strategies originating from Chinese directly conflict with the morphology-based strategy from Russian within a single sentence, the real-time mechanisms in the learner’s brain—their attentional allocation, consumption of cognitive resources, and ultimate decision-making path—remain largely unknown.

To this end, the present study investigates this issue by employing eye-tracking methodology. We examine how native Chinese-speaking learners of Russian weigh different cues in real time when confronted with referential ambiguity. By comparing two proficiency groups, the study asks whether L2 proficiency is associated with differences in ambiguity-processing efficiency and in the allocation of attention across competing antecedents. Overall, this comparison provides evidence on how cue integration during real-time reference resolution changes with L2 proficiency in Russian, and on how L2 learners weigh and coordinate competing referential cues during real-time sentence processing.

## 4. Materials and Methods

### 4.1. Participants

A total of 49 native Chinese-speaking learners of Russian were recruited for this study. Following recommendations for transparent sample-size justification [43], the planned sample size was informed by two considerations. First, comparable eye-tracking and visual-world studies of L2 anaphora resolution have typically used between 16 and 24 participants per proficiency or language group, with main effects of ambiguity and proficiency reliably detected at these sample sizes, e.g., [24,26,44,45]. Our target of 23 participants per proficiency group is consistent with this range. Second, to characterize what the present design can support inferentially, we conducted a sensitivity power analysis using G*Power 3.1.9.7. Given the final sample of 46 participants, α = 0.05, and a target power of 0.80, the 2 × 2 mixed design was sensitive to detect within-subjects main effects and within × between interaction effects as small as f ≈ 0.21, and between-subjects main effects as small as f ≈ 0.37, corresponding to small-to-medium effects by Cohen’s benchmarks [46]. All effects of theoretical interest in the present study exceeded these thresholds (see Section 5); where individual estimates approached the sensitivity bound, we treat them with corresponding caution in interpretation. Three participants—two from the high-proficiency group and one from the low-proficiency group—were excluded because eye-tracking data could not be successfully collected due to excessive myopia or astigmatism, or a history of eye surgery. The final sample therefore comprised 46 participants, divided into a high-proficiency group (*n* = 23, M_age = 22.96 years, SD = 1.07; 8 males, 15 females) and a low-proficiency group (*n* = 23, M_age = 19.78 years, SD = 0.52; 7 males, 16 females).

All participants were ab initio learners of Russian, were right-handed, and had normal or corrected-to-normal vision, with no history of neurological or reading disorders. The study was approved by the university’s ethics committee, and all participants provided written informed consent before the experiment and received remuneration upon completion.

The low-proficiency group consisted of second-year university undergraduates who had all passed the Test for Russian Majors—Band 4 (TEM-4). The high-proficiency group consisted of master’s students who had all passed the Test for Russian Majors—Band 8 (TEM-8), which represents the highest proficiency level for undergraduate Russian majors. To validate the effectiveness of this grouping, an independent samples *t*-test was conducted on key data from the language background questionnaire. The results showed that the high-proficiency group’s self-rated Russian proficiency scores (M = 5.96, SD = 0.77) were significantly higher than those of the low-proficiency group (M = 3.91, SD = 0.59), t(44) = 10.08, *p* < 0.001. The high-proficiency group also reported significantly greater daily language exposure and higher self-ratings across multiple language skills (all ps < 0.01). These results confirm that the two groups differed reliably in both Russian proficiency and degree of language exposure.

### 4.2. Experimental Design

The experiment employed a 2 × 2 mixed design. The two factors were Sentence Type (referentially ambiguous vs. referentially unambiguous), a within-subjects factor, and Participant Proficiency (high vs. low), a between-subjects factor.

### 4.3. Materials

The experimental materials were developed by adapting and rigorously screening the sentences used in Yurchenko [47]. To ensure their suitability and methodological rigor, we carried out a series of modifications followed by multiple rounds of pretesting.

First, we systematically controlled the vocabulary used in the original 160 sentences. The frequency, length, and predictability of all lexical items were balanced and optimized with reference to relevant corpora. Given the linguistic proficiency of the lower-proficiency participants, any word that exceeded the vocabulary syllabus of the Test for Russian Majors—Band 4 (TEM-4) was replaced with a synonym of moderate difficulty, for example, replacing *polkovnik* ‘colonel’ with *soldat* ‘soldier’, while keeping the core semantics and sentence structure unchanged.

After the vocabulary modifications were completed, the materials were screened through three stages of pretesting:

(1) Plausibility rating by native Russian speakers. Forty-four native Russian speakers rated the plausibility of the 160 adapted sentences on a seven-point scale. Only sentences with a mean rating above 6.0 were retained for subsequent screening.
(2) Ambiguity rating by native Russian speakers. For the sentences that passed the plausibility screening, the same group of native speakers answered a forced-choice question (*kto?* ‘who?’) targeting the ambiguous pronoun. To ensure genuine referential ambiguity, we retained only those sentences for which the proportions of antecedent choices fell between 40% and 60%. This criterion ensured that the two potential antecedents were considered nearly equally likely by native speakers.
(3) Vocabulary familiarity rating by L2 learners. To ensure that the experimental materials were comprehensible to the target participant population, we recruited 22 learners with approximately one year of Russian study who did not take part in the main experiment. They rated the familiarity of all words in the sentences that had passed the first two screening rounds on a seven-point scale. Only sentences whose component words had mean familiarity scores above 5.65 were retained, ensuring that the participants would have sufficient understanding of both the lexical items and the sentence meanings.

Following the screening process described above, we finalized a set of 62 structurally parallel sentences to serve as the core stimuli for the experiment. From this set, we created two experimental conditions:

(1) Ambiguous condition. The 62 original sentences that passed all screening stages.
(2) Unambiguous condition (morphologically disambiguated condition). These sentences were created by modifying the gender of the second antecedent in the ambiguous sentences so that it no longer matched the grammatical gender of the subsequent pronoun *on* (‘he’), thus formally eliminating the ambiguity.

The experiment therefore consisted of 62 sentence structures, each instantiated in two conditions, yielding 124 experimental sentences. Using a blocked random design, we distributed these sentences into two experimental lists (List A and List B). Each list contained 62 sentences, ensuring that each structure appeared only once and that the number of items per condition was balanced. Each participant was exposed to only one list, and participants from the two proficiency groups were evenly assigned to the lists. In addition, 62 filler sentences, which were structurally similar but contained only one unambiguous subject, were added to each list. All experimental and filler sentences were presented in a pseudorandom order.

### 4.4. Apparatus

Eye movements were recorded using a Tobii Spectrum eye-tracker (Tobii Pro AB, Danderyd, Sweden) at a sampling rate of 1200 Hz, with a manufacturer-reported spatial accuracy of approximately 0.3° and precision of approximately 0.06° RMS under typical recording conditions. Stimuli were presented on a 23-inch HP ENVY 23 monitor (HP Inc., Palo Alto, CA, USA; active display area approximately 509 × 286 mm, resolution 1920 × 1080 pixels, refresh rate 60 Hz). The experimenter monitored the session from a separate computer. The programming of the experiment, stimulus presentation, and initial data processing were carried out using Tobii Pro Lab, version 1.232.52429. Sentences were presented in a single line in Times New Roman, 48 pt, with 1.15 line spacing, as black text on a white background. The stimulus display area used 5% screen margins. At a viewing distance of 60–65 cm (see Section 4.5), the height of a single character corresponded to approximately 1.5° of visual angle, and a single character occupied approximately 0.5–0.7° horizontally depending on letter width.

Areas of interest (AOIs) were defined a priori around the first antecedent noun phrase (A1), its immediate spillover region (B1), the second antecedent noun phrase (A2), its immediate spillover region (B2), the critical pronoun region (C, corresponding to the pronoun *on* ‘he’), and the post-pronoun spillover region (S). AOIs were defined on the basis of word boundaries in each sentence; the antecedent regions corresponded to the two potential referential candidates, and the spillover regions to the words immediately following them. All AOIs comfortably exceeded the spatial accuracy of the Tobii Spectrum, ensuring reliable assignment of fixations to the intended regions.

### 4.5. Procedure

After providing written informed consent, participants were seated approximately 60 to 65 cm from the monitor. A standard five-point eye-tracking calibration was then performed. The experiment proceeded only after a calibration error of less than 0.5° was achieved; otherwise, the calibration was repeated. Drift correction was performed before each experimental block, and recalibration was conducted whenever the experimenter observed unstable tracking or poor gaze-position accuracy. All procedures were approved by the university’s ethics committee.

Fixations and saccades were identified in Tobii Pro Lab using the default Tobii I-VT (Fixation) filter. This velocity-threshold algorithm classifies a gaze sample as belonging to a fixation when the eye-movement velocity is below 30°/s, and as belonging to a saccade when it exceeds 30°/s. The minimum fixation duration was set to the Tobii Pro Lab default of 60 ms; adjacent fixations separated by less than 75 ms and falling within 0.5° of visual angle were merged according to the default I-VT adjacency settings. The default I-VT settings were retained because the experiment used a screen-based reading task with relatively stable head position, and no additional acceleration-based threshold was applied. Trials were excluded from the analysis if they exhibited substantial track loss (more than 25% of the trial duration without valid gaze data), prolonged blinks, or systematic data loss in critical AOIs. Across all participants, 1.9% of trials were excluded for these reasons, leaving approximately 60.8 trials per participant on average for analysis. To verify that the automatic fixation classification accurately captured participants’ reading behavior, a randomly selected 10% of trials were visually inspected by the first author against the raw gaze samples. No systematic misclassifications between fixations and saccades were identified, and the inspected trials were consistent with the algorithmic output.

The main experiment was preceded by five practice trials. Each trial began with a central fixation cross. Once a stable fixation was detected, a single-line sentence, either experimental or filler, appeared on the screen. Participants read the sentence at their own pace and pressed the spacebar to continue. Each sentence was followed by a two-alternative forced-choice comprehension question, which participants answered using a mouse click.

To ensure participant attentiveness, 62 filler sentences were included. Each filler question contained one correct answer and one distractor. Data from any participant whose accuracy on these filler trials fell below 83.3 percent were excluded from the final analysis.

### 4.6. Data Preprocessing and Statistical Analysis

All statistical analyses were conducted in R version 4.5.1 using the dplyr, readxl, lme4, and lmerTest packages. Sum contrasts were used for categorical predictors. Reaction times and continuous eye-movement duration measures were log-transformed before model fitting.

Linear mixed-effects models were fitted to continuous dependent variables, including response times, whole-sentence total reading durations, AOI total gaze durations, and AOI first-pass durations. Generalized linear mixed-effects models were fitted to binary referential-choice data using a binomial link function and to fixation-count data using a Poisson distribution. Fixed effects included Proficiency Group, Sentence Condition, and their interaction; the antecedent-region AOI model additionally included Antecedent Position and its interactions with the other factors. All models included random intercepts for participants and items. Random slopes were not included to maintain model convergence and interpretability. Degrees of freedom and *p* values for linear mixed-effects models were obtained using the Satterthwaite approximation implemented in lmerTest.

## 5. Results and Analysis

This study collected both offline and online measures to investigate the cognitive processes and referential choices of L2 learners at different proficiency levels during ambiguity resolution. Offline measures included reaction times on the judgment task and final referential choice preferences. Online eye-tracking measures were divided into whole-sentence measures and region-of-interest measures. Whole-sentence measures included total reading duration and total fixation count. Region-of-interest measures included first-pass duration, defined as the summed duration of fixations from first entering an AOI until first leaving it, and total gaze duration, defined as the summed duration of all fixations within an AOI. The Supplementary Materials for this article are available online.

### 5.1. Offline Behavioral Data Analysis

We first analyzed the offline behavioral data, which included reaction times on the judgment task and the final referential choice preferences. All descriptive statistics are detailed in Table 1.

#### 5.1.1. Reaction Times on the Judgment Task

To assess the cognitive effort involved in the referential judgment task, we fitted a linear mixed-effects model to the reaction time data after removing outliers. The analysis revealed no significant interaction between proficiency and sentence condition (β = 0.016, SE = 0.009, t(2683) = 1.69, *p* = 0.091). There was, however, a significant main effect of proficiency (β = −0.11, SE = 0.05, t(44) = −2.07, *p* = 0.044). The main effect of sentence condition was not significant (*p* = 0.157).

#### 5.1.2. Referential Choice Preferences

**Figure 1:**
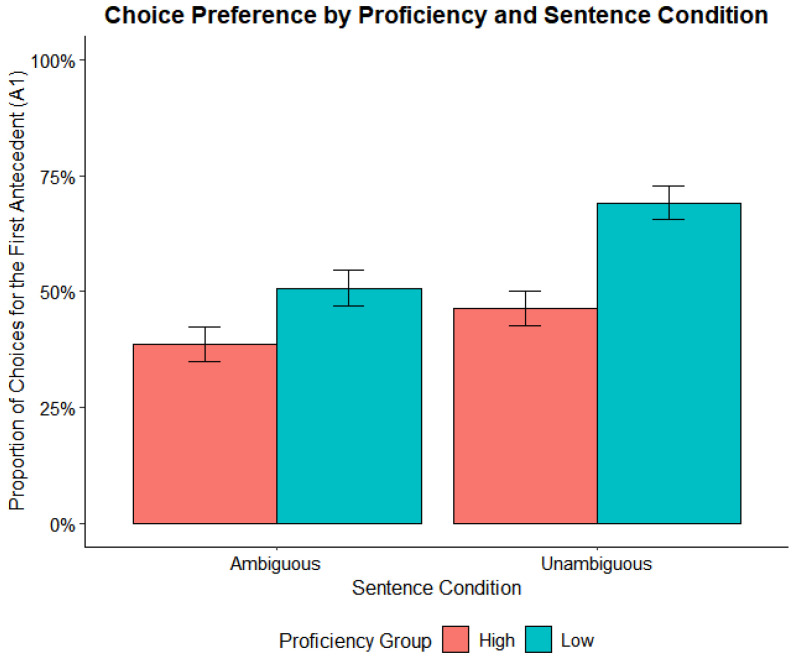
Proportion of choices for the first antecedent (A1) as a function of Sentence Condition and Proficiency Group. Error bars represent 95% confidence intervals.

To examine the learners’ referential choices, we fitted a Generalized Linear Mixed-Effects model to the choice data. The model revealed the significant main effects of proficiency (β = 0.40, SE = 0.09, z = 4.36, *p* < 0.001) and sentence condition (β = 0.30, SE = 0.04, z = 7.04, *p* < 0.001). Crucially, the model also revealed a highly significant interaction between the two factors (β = −0.12, SE = 0.04, z = −2.93, *p* = 0.003) (see Figure 1).

### 5.2. Online Eye-Tracking Data Analysis

#### 5.2.1. Global Reading Measures

Descriptive statistics for whole-sentence eye-tracking measures are presented in Table 2.

**Figure 2:**
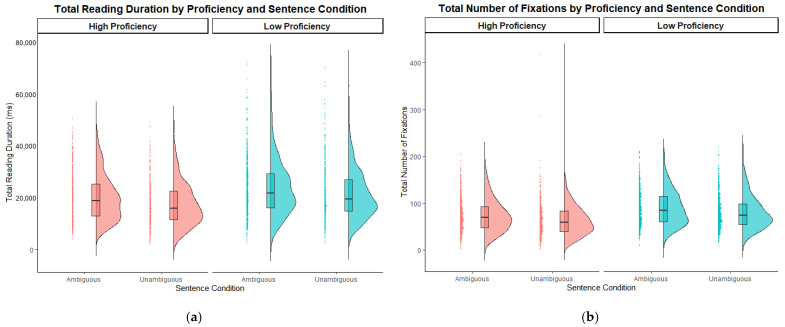
(**a**) Raincloud plot of total reading duration as a function of Sentence Condition, faceted by Proficiency Group; (**b**) Raincloud plot of total number of fixations as a function of Sentence Condition, faceted by Proficiency Group.

For the analysis of total sentence reading time, we fitted a Linear Mixed-Effects model with proficiency, sentence condition, and their interaction as fixed effects, and with subjects and items as random effects. The results revealed a significant interaction between proficiency and sentence condition (β = 0.017, SE = 0.007, t(2600) = 2.24, *p* = 0.025; see Figure 2a). The model also showed significant main effects of both sentence condition (β = 0.064, SE = 0.007, t(2561) = 8.89, *p* < 0.001) and proficiency (β = −0.098, SE = 0.042, t(43) = −2.35, *p* = 0.023).

To assess the depth of processing while reading the sentence, we conducted a Generalized Linear Mixed-Effects model on the fixation-count data, using a Poisson distribution appropriate for count variables. Consistent with the results for total reading time, there was a significant main effect of sentence condition (β = 0.061, SE = 0.002, z = 27.43, *p* < 0.001), indicating that participants made more fixations on ambiguous sentences than on unambiguous ones. The main effect of proficiency was likewise significant (β = −0.111, SE = 0.040, z = −2.77, *p* = 0.006), and there was a significant interaction between the two factors (β = 0.009, SE = 0.002, z = 4.12, *p* < 0.001; see Figure 2b).

#### 5.2.2. Area of Interest (AOI) Analysis

**Figure 3:**
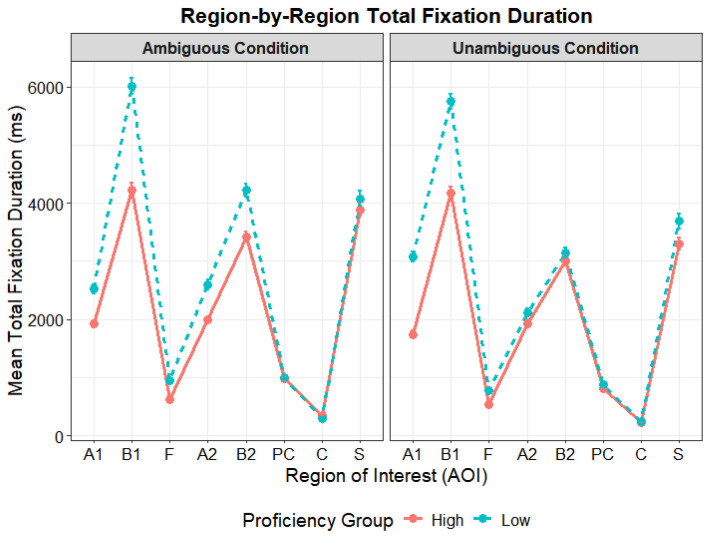
Mean total fixation duration (in ms) on each region of interest (AOI) as a function of Sentence Condition, plotted separately for each Proficiency Group.

An overview of mean total fixation duration across the regions of interest, by sentence condition and proficiency group, is shown in Figure 3.

##### Antecedent (A1, A2) and Spillover (B1, B2) Regions

In the antecedent and spillover regions preceding the pronoun, an initial analysis revealed a main effect of proficiency on both total reading time and first-pass duration in the A1 and A2 regions (ps < 0.05). To examine whether antecedent position, that is, first-mention versus second-mention, modulated this pattern, we fitted a linear mixed-effects model that included Antecedent Position, Sentence Condition, and Proficiency as fixed factors. This model revealed a significant three-way interaction among the three factors (β = 0.039, SE = 0.009, t = 4.10, *p* < 0.001).

To interpret this interaction and to examine the processing patterns of each proficiency group in the baseline unambiguous condition, we conducted targeted follow-up analyses. These analyses showed that when reading unambiguous sentences, the low-proficiency learners displayed a strong first-mention bias. Their total gaze duration on the first antecedent phrase (A1 + B1) was substantially longer than that on the second antecedent phrase (A2 + B2) (β = 0.69, SE = 0.04, t(1224) = 19.18, *p* < 0.001), indicating a “sentence-initial anchoring” strategy. In addition, compared with the high-proficiency learners, the low-proficiency learners spent significantly more time processing the first antecedent phrase (A1 + B1) (β = 0.54, SE = 0.16, t(43.8) = 3.46, *p* = 0.001), suggesting reduced processing efficiency.

**Figure 4:**
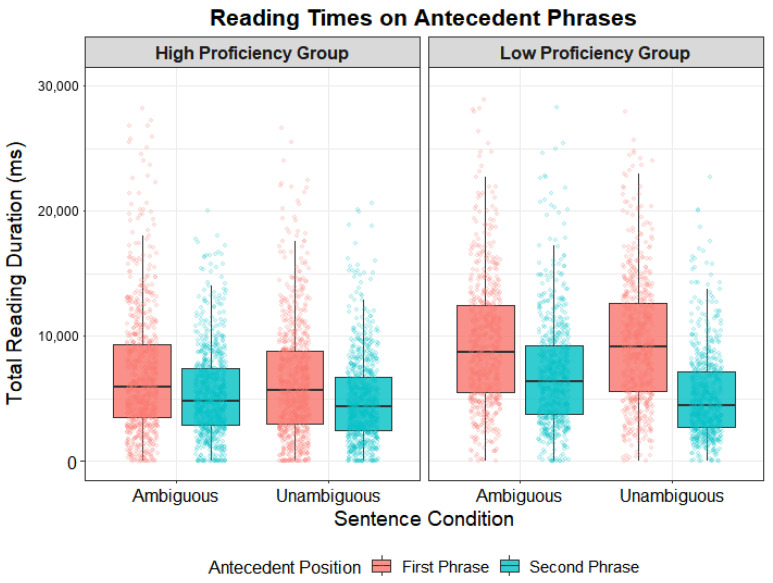
Boxplots showing the distribution of total reading duration on the first and second antecedent phrases. Data are plotted by Sentence Condition and faceted by Proficiency Group.

The distribution of total reading duration across the first and second antecedent phrases is shown in Figure 4.

##### Critical Region (Region C: The Pronoun on)

When participants reached the critical pronoun *on*, an ambiguity effect emerged immediately. The analysis revealed a significant main effect of sentence condition on both total gaze duration (*p* < 0.001) and first-pass duration (*p* < 0.05). These results indicate that, regardless of proficiency, all participants experienced immediate processing difficulty when encountering the pronoun in ambiguous sentences, as reflected in longer gaze durations.

##### Spillover Region (Region S)

In the spillover region following the pronoun, the processing difficulty triggered by the ambiguity persisted. The analysis of total gaze duration again showed a significant main effect of sentence condition (*p* < 0.001). This pattern represents a classic spillover effect, indicating that the processing difficulty encountered at the critical region carried over into the subsequent text. No significant effects were found for first-pass duration.

## 6. Discussion

### 6.1. The Relationship Between Language Proficiency and Processing Difficulty

A primary finding of this study is that, for L2 learners, processing sentences containing referential ambiguity requires greater cognitive effort than processing morphologically disambiguated sentences. Online eye-tracking data from global reading measures show that total reading time was significantly longer for ambiguous sentences than for disambiguated sentences (significant main effect of sentence condition, *p* < 0.001), and that participants made significantly more fixations when reading ambiguous sentences (significant main effect of sentence condition, *p* < 0.001). This finding confirms that ambiguity resolution is a complex process that consumes cognitive resources. Increases in reading time and fixation counts are widely considered direct indicators of heightened cognitive load [48,49,50]. When the processing system encounters a pronoun that could refer to multiple antecedents, it must perform additional computations, such as maintaining multiple possible syntactic analyses in working memory or engaging in syntactic reanalysis when an initial judgment proves incorrect. These additional computational steps slow reading and require more detailed visual information intake (i.e., more fixations), leading to the observed ambiguity effect. The findings of this study are broadly consistent with a large body of research on both native speakers [11,51,52] and other groups of L2 learners [26,44,45]. This suggests that the learners of Russian in our study, like language users generally, are highly sensitive to the structural complexity introduced by referential ambiguity and pay a corresponding cognitive cost.

Furthermore, we found that language proficiency plays a crucial role in modulating processing efficiency. The data show that high-proficiency learners significantly outperformed low-proficiency learners in overall processing. Their efficiency was higher both in the offline task that required a final judgment (significantly faster overall reaction times, *p* = 0.044) and during the more natural online reading process (significantly shorter total reading times, *p* = 0.023). Most importantly, they comprehended the sentences with significantly fewer fixations (significant main effect of total fixation count, *p* = 0.006), suggesting a higher level of efficiency in information acquisition and integration. For low-proficiency learners, many fundamental linguistic operations—such as lexical retrieval, lexical access, and basic syntactic parsing—are far from being automated. These operations consume substantial attentional and working memory resources, leaving limited residual resources for higher-level, more complex tasks such as resolving syntactic ambiguity.

As proficiency increases, this situation changes. Long-term language exposure and use lead to the high-level automaticity of these foundational language skills. This process of automatization means that these operations can be executed quickly and efficiently, with minimal demands on attentional and working-memory resources. This “liberation” of cognitive resources is a key factor underlying the greater processing efficiency observed among high-proficiency learners [53]. Once learners no longer expend substantial resources on lexical retrieval and basic syntactic parsing, they can allocate more cognitive capacity to the core demands of the task, namely evaluating, weighting, and integrating multiple competing antecedents. Thus, the faster reading times and reduced fixation counts observed in high-proficiency learners are not simply superficial indicators of “increased reading speed”. Rather, they represent behavioral manifestations of a more optimized system of resource allocation. A more automated lower-level processing system provides “the computational capacity” necessary to support the higher-level operations involved in resolving referential ambiguity. The data from this study clearly illustrate how this difference in processing efficiency narrows as proficiency increases.

### 6.2. Proficiency-Related Processing Strategies and Cue Integration

A second major finding concerns the processing strategies adopted by low-proficiency learners. In the offline task with morphologically disambiguated sentences, when the grammatical cue of gender agreement favored the first antecedent (A1), low-proficiency learners selected that antecedent 69.1 percent of the time, significantly more often than high-proficiency learners, who did so 46.4 percent of the time (significant interaction, *p* = 0.003). The online eye-tracking data corroborated this pattern. When reading morphologically disambiguated sentences, low-proficiency learners devoted substantially more total gaze time to the first antecedent phrase (A1 + B1) than to the second (A2 + B2) (*p* < 0.001). Taken together, these results point to a robust first-mention bias among low-proficiency learners, while also suggesting that the Russian gender cue was not used uniformly across participants.

This pattern can be understood in relation to how discourse prominence is encoded across the learners’ L1 and L2. According to Centering Theory [54], discourse coherence depends on maintaining an attentional center, typically associated with the most prominent entity in the preceding discourse. Languages differ in the cues that contribute to this prominence. In English, grammatical subjecthood and sentence-initial position often converge. In Chinese, discourse topics can appear sentence-initially and guide coherence across clauses [55]. Russian presents a different configuration: rich nominal morphology allows grammatical roles to be identified through case and agreement marking, while relatively flexible word order can also contribute to information-structural and pragmatic interpretation. For Chinese-speaking learners of Russian, this creates a processing context in which position-based prominence and morphosyntactic agreement cues must be coordinated during real-time reference resolution.

The first-mention pattern observed in the low-proficiency group is consistent with previous findings that L2 learners often associate a pronoun with the first-mentioned noun phrase in a sentence, e.g., [56,57]. Across both pro-drop and non-pro-drop languages, an antecedent’s subjecthood and first-mention status can enhance its discourse prominence [58,59]. Similar patterns have also been reported by Fujita and Cunnings [60]. In the present study, low-proficiency learners appear to have relied heavily on the earliest prominent antecedent, especially when this antecedent was compatible with the available morphosyntactic information. During incremental sentence processing, such a strategy may reduce processing demands: once an early plausible antecedent is identified, fewer resources are allocated to evaluating later candidates. This account is consistent with the broader view that L2 learners under processing pressure may depend on relatively accessible discourse cues when full integration of morphosyntactic and discourse information is difficult.

The high-proficiency group showed a markedly different profile. They read more quickly overall and made fewer fixations, suggesting greater processing efficiency. However, their offline choices were notable: in the morphologically disambiguated condition, where the gender cue licenses only the first antecedent (A1), high-proficiency learners selected A1 only 46.4% of the time—a rate at or near chance—while low-proficiency learners selected A1 69.1% of the time. That high-proficiency learners did not preferentially follow the gender cue, despite reading more efficiently, is the central interpretive challenge of the present data. Several non-exclusive accounts are compatible with this pattern. First, high-proficiency learners may be engaging in efficient but partially resolved reference processing: they read quickly because they tolerate unresolved reference rather than because they commit to a gender-licensed interpretation. Second, the pattern may reflect persistent difficulty in deploying Russian gender agreement in real-time reference resolution, consistent with the broader observation that morphosyntactic cues, although formally reliable, are not always exploited by L2 readers under time pressure. Third, the near-chance offline choices may reflect decision-level uncertainty or inability to commit, distinct from the online processing operations indexed by reading times. The present design cannot fully distinguish among these accounts, but we note that all three share a substantive prediction: even advanced Chinese-speaking learners of Russian do not categorically integrate gender agreement into reference resolution, and the proficiency advantage observed here lies primarily in processing efficiency rather than in cue accuracy. The difference between the two groups is therefore best characterized as a proficiency-related shift in cue weighting and resource allocation: low-proficiency learners relied more strongly on first-mention prominence, while high-proficiency learners showed faster processing without categorically committing to the morphologically licensed antecedent.

Across all three accounts, a parsimonious reading is that the referential task itself imposed integration demands that the present participants—including advanced learners—could not fully meet within the available reading time. We do not commit to a more specific cognitive explanation of why these demands exceeded learners’ capacity; distinguishing among the accounts above awaits work with native Russian baselines, larger samples, and tasks that decompose the components of referential integration.

A third key finding concerns the processing of the critical region, containing the ambiguous pronoun *on* (Region C). We observed a significant main effect of sentence condition on both first-pass duration (*p* < 0.05) and total gaze duration (*p* < 0.001). This indicates that, regardless of proficiency, all participants experienced immediate processing difficulty when encountering the pronoun in ambiguous sentences.

Research on native speakers has shown that the detection of referential ambiguity is highly incremental [61,62,63]. Comprehenders do not wait until the end of a sentence to begin integration and interpretation; rather, they attempt to incorporate each word into an evolving syntactic and semantic representation as it is encountered. The present findings suggest that L2 learners also detected the referential conflict at the point of the pronoun, as reflected in immediate slowdown. This pattern supports accounts proposing that L2 processing can share core incremental mechanisms with L1 comprehension [64,65,66], even when learners differ in how efficiently they resolve the conflict.

The processing difficulty was not confined to the critical pronoun. In the post-pronoun spillover region (Region S), total gaze duration was again significantly longer in ambiguous sentences than in morphologically disambiguated sentences (*p* < 0.001). This spillover effect suggests that the ambiguity-related processing initiated at the pronoun region was not fully resolved within that region. Instead, unresolved operations such as maintaining multiple antecedent candidates, evaluating cue compatibility, and attempting referential integration continued into the following text, resulting in longer reading times in the spillover region.

## 7. Limitations and Future Directions

A core contribution of this study lies in its focus on the typologically distinct Chinese–Russian language pair, a combination that makes it possible to observe proficiency-related differences in L2 ambiguity resolution. At the same time, this specificity also limits the generalizability of the findings. Because the present design included only Chinese-speaking learners of Russian and did not include a native Russian control group, the results should be interpreted as proficiency-related differences within L2 Russian rather than as evidence of native-like or non-native-like resolution strategies. Future studies should include an L1 Russian baseline and examine additional typologically distant language pairs, such as Chinese-Arabic or English-Japanese, to determine how broadly the observed processing patterns generalize.

The morphologically disambiguated condition also requires caution. Because this condition was created by manipulating grammatical gender, it was not a neutral baseline but a condition that actively supplied a gender-agreement cue. The fact that learners did not always select the antecedent licensed by this cue suggests that the use of Russian gender morphology in real-time reference resolution remains difficult, even for advanced learners. This difficulty is theoretically informative and should be examined more directly in future work.

The cross-sectional design further limits the interpretation of proficiency-related differences. Although comparing high- and low-proficiency learners reveals clear group differences, such a design does not capture within-learner developmental change. Longitudinal work following the same cohort over time would provide a more detailed understanding of how processing strategies evolve as proficiency increases.

Finally, the experimental materials impose limits on ecological validity. The use of highly controlled individual sentences ensured strong internal validity, but naturalistic discourse provides richer contextual cues that may modulate the strategies learners employ. Future studies could therefore investigate whether the processing patterns observed here extend to more natural discourse contexts, such as passage reading.

## 8. Conclusions

To our knowledge, this study is among the first to use eye-tracking to examine how Chinese learners of Russian process referential ambiguity during reading. The findings show that ambiguous pronouns imposed a measurable online processing cost for both proficiency groups, indicating that referential ambiguity remains cognitively demanding in L2 Russian.

Proficiency modulated how learners managed this difficulty. Low-proficiency learners showed a stronger first-mention anchoring pattern, suggesting greater reliance on the earliest prominent antecedent. High-proficiency learners read more efficiently overall and showed less processing disruption, although their offline responses indicate that the use of Russian gender agreement as a referential cue was not fully stable. Increased proficiency therefore appears to support more efficient cue coordination and resource allocation during reference resolution.

Taken together, the data suggest that referential ambiguity remained cognitively demanding for both proficiency groups, with the high-proficiency group performing only marginally better; the proficiency advantage observed here is one of efficiency, not of categorical cue use.

The two proficiency groups differed primarily in processing efficiency and the relative weighting of competing referential cues, rather than in whether they detected the ambiguity or used a single dominant strategy. High-proficiency learners read more efficiently overall, but their offline responses showed that they did not categorically commit to the gender-licensed antecedent in the disambiguated condition. This pattern is compatible with several non-exclusive interpretations: more efficient but uncertain resolution, persistent difficulty in using Russian gender morphology under time pressure, or decision-level processes that tolerate unresolved reference. Even so, the findings indicate that proficiency-related variation in L2 reference resolution involves changes in efficiency and cue weighting that are measurable at the level of online eye movements. Distinguishing among these accounts requires further work, ideally with native Russian baselines. Taken together, these findings deepen our understanding of ambiguity resolution in the Chinese–Russian language pair and demonstrate the value of eye-tracking for separating immediate ambiguity detection from later resolution processes. Future studies with native-speaker baselines, larger samples, and longitudinal designs will help clarify how proficiency-related change in L2 sentence processing involves shifts not only in accuracy but also in the strategic allocation of cognitive resources across competing cues.

## Figures and Tables

**Table 1:** Descriptive Statistics for the Offline Comprehension Task.

Proficiency Group	Sentence Condition	Choice Proportion for Antecedent 1 (%)	Response Time (ms) M (SD)
High	Ambiguous	38.7	1719 (948)
	Unambiguous	46.4	1677 (953)
Low	Ambiguous	50.8	2140 (1169)
	Unambiguous	69.1	2184 (1217)

**Table 2:** Descriptive Statistics for Whole-Sentence Eye-Tracking Measures.

Proficiency Group	Sentence Condition	Total Reading Duration (ms)	Total Fixation Count
High	Ambiguous	19,972	73.5
	Unambiguous	17,335	64.2
Low	Ambiguous	23,629	89.3
	Unambiguous	21,534	80.2

## Data Availability

The datasets generated and analyzed during the current study are available in the Open Science Framework repository, https://osf.io/4e25a/overview?view_only=d07dfa17226944c08877b376bfab1ce6 (accessed on 1 June 2026).

## Supplementary Materials

The following supporting information can be downloaded at: https://osf.io/4e25a/overview?view_only=8126b0fcc8ab40efa0a19d9c83c5608a (accessed on 1 June 2026).

## Abbreviations

The following abbreviations are used in this manuscript:

L1	First Language
L2	Second Language
AOI	Area of Interest
